# Detection of surface defects in soybean seeds based on improved Yolov9

**DOI:** 10.1038/s41598-025-92429-3

**Published:** 2025-04-12

**Authors:** Chuanming Liu, Yifan Shen, Feng Mu, Haixia Long, Anas Bilal, Xia Yu, Qi Dai

**Affiliations:** 1 Key Laboratory of Data Science and Smart Education, Hainan Normal University, Ministry of Education, Haikou, 571158 China; 2 School of Information Science and Technology, Hainan Normal University, Haikou, 571158 China; 3 Affiliation College of Life Science and Medicine, Zhejiang Sci-Tech University, Hangzhou, China

**Keywords:** Yolov9, Soybean seeds, Computer vision, Surface defect detection, Plant breeding, Plant reproduction

## Abstract

As one of the important indicators of soybean seed quality identification, the appearance of soybeans has always been of great concern to people, and in traditional detection, it is mainly through the naked eye to check whether there are defects on its surface. The field of machine learning, particularly deep learning technology, has undergone rapid advancements and development, making it possible to detect the defects of soybean seeds using deep learning technology. This method can effectively replace the traditional detection methods in the past and reduce the human resources consumption in this work, leading to decreased expenses associated with agricultural activities. In this paper, we propose a Yolov9-c-ghost-Forward model improved by introducing GhostConv, a lightweight convolutional module in GhostNet, which enhances the recognition of soybean seed images through grayscale conversion, filtering processing, image segmentation, morphological operations, etc. and greatly reduces the noise in them, to separate the soybean seeds from the original images. Based on the Yolov9 network, the soybean seed features are extracted, and the defects of soybean seeds are detected. Based on the experiments’ findings, the recall rate can reach 98.6%, and the mAP0.5 can reach 99.2%. This shows that the model can provide a solid theoretical foundation and technical support for agricultural breeding screening and agricultural development.

## Introduction

As a critical crop globally, soybean has become one of the most traded commodities^1^ and is one of the major crops grown worldwide that affects different aspects of the ecosystem^2^. It is the most important seed legume globally, accounting for 25 per cent of the world’s total edible oil production, and plays an important role as the main source of protein concentrate used for livestock nutrition for about two thirds of the world’s livestock^3^. Soybeans have seed quality directly related to final yield and quality. Seed surface defects are among the most important indicators for assessing quality^4^. Still, soybean seeds are highly susceptible to external factors such as climate, mechanical damage, pests, and diseases during seed production, storage, and transportation, resulting in various surface defects^5^. These seed defects pose a severe threat to agricultural production. They can increase the risk of pests and diseases, lead to the excessive use of pesticides and fertilizers, and cause environmental pollution. Therefore, developing advanced soybean seed surface defect detection technology is of far-reaching significance in reducing the use of pesticides and chemical fertilizers, reducing the negative impact of agricultural production on the environment, and promoting agriculture’s green and sustainable development^6^.

Traditional soybean seed quality testing methods have relied heavily on human labor. These methods include field planting identification^7^, chromatographic analysis techniques^8^, inspection by naked eyes, and measurement with traditional tools such as vernier calipers and electronic scales.. These methods are not only time-consuming and labor-intensive but also do not readily form a common standardized scheme and are easily affected by subjective factors, resulting in inconsistent and inefficient test results from different inspectors in different regions.

As the field of artificial intelligence grows, deep learning methods have made breakthroughs in areas such as medical image analysis, autonomous driving, natural language processing, and game AI. Hence, this thesis advocates for leveraging deep learning to detect surface defects in soybean seeds, enhancing detection efficiency and accuracy in agriculture, enhancing the quality of screened seeds, and ultimately fostering the modernization and intelligent progression of global agricultural production.

Tian et al. reviewed the practical applications of computer vision in agriculture, highlighting its role in automating small farm processes for cost-effective and precise results. Their focus, however, was on automation rather than the technical details of computer vision^9^.

Researchers have explored image processing and computer vision to address challenges in plant science and agriculture. Dokic et al. found deep-learning methods superior to classical machine-learning methods for crop classification. However, high-density scenes remain inadequately addressed, and the evaluation of deep neural networks in agriculture is still in its early stages, lacking widespread attention^10^.

Osorio et al. achieved notable weed classification accuracies using advanced models like Mask R-CNN, Yolov3, and SVM, with F1 scores of 94%, 88%, and 94%, respectively. Their results demonstrated that deep learning models enhance weed cover estimation accuracy while reducing human bias^11^. Yu et al. explored various DCNN models for Bermudagrass detection, with VGGNet achieving the highest F1 score (> 0.95), outperforming GoogLeNet in weed detection tasks^12^. They also proposed strategies to improve the precision of deep learning models. However, weed detection alone is insufficient for yield improvement; identifying crops is equally critical to boost overall productivity. Gao et al. refined Yolov3 and Tiny Yolo architectures, achieving detection accuracies of 0.761 for cornflower and 0.897 for sugar beet using 800 × 1200 pixel images^13^. While this redesign improved speed and accuracy, practical applications may still face challenges due to information loss and occasional misjudgments.

Lin et al. introduced a CNN model to precisely detect diverse symptoms and progression stages of a particular grape disease, facilitating targeted diagnosis and management. Detecting plant diseases using object detection techniques saves practitioners’ time and effort and allows for real-time judgments. This helps practitioners take timely interventions and control measures on the plants. By doing so, they effectively curb the spread of diseases and minimize the detrimental effects on crop yields and quality.^14^. In another study, Wang et al. employed a modified YoloV5s model, focusing on channel-based optimizations, to swiftly detect apple fruits, enabling early yield estimation^15^. Meanwhile, Sozzi et al. automated the identification of grape bunches from white grape varieties by leveraging multiple iterations of the Yolo model. Their experimental outcomes provided valuable insights into the accuracy and efficiency of various detection techniques in practical applications^16^. Khaki et al. successfully detected and counted corn kernels in images utilizing CNNs. Training various models enabled high-speed object detection across diverse lighting conditions and angles.

Additionally, they employed the conventional sliding window technique to detect corn kernels, achieving remarkable accuracy^17^. On the other hand, Cardellicchio et al. leveraged the YoloV5 model to identify surface features of tomato plants, enabling monitoring of their growth process and facilitating yield predictions based on the detection outcomes^18^. These studies demonstrate the feasibility of applying computer vision and deep learning techniques to detect agricultural products.

Various methods and techniques in agricultural weed recognition are described in a review by Rakhmatulin et al. The article shows deep-learning models perform better than traditional computer vision methods^19^.

In the field of detecting soybean seed defects using deep learning methods, Pratap et al. used three models, VGG16, AlexNet and CNN, to classify and identify the quality of soybean seeds, with accuracies of 90%, 78.49% and 74.55%, respectively^20^.

SAITO et al. used a multi-input convolutional neural network with colour and UV fluorescent image inputs to detect external soybean seed SAITO et al. used multi-input convolutional neural networks with colour and UV fluorescence image inputs to detect external defects of soybean seeds, and constructed a multi-input CNN model using three pre-trained network models, including AlexNet, ResNet-18, and EfficientNet, of which the multi-input CNN model using ResNet-18 had the highest accuracy rate of 93.9%. Both methods have their merits, but the accuracy is not high enough and still causes large losses in practical applications^21^.

As one of the culprits leading to a sharp decrease in soybean yield, Asian Soybean Rust (ASR) has a huge impact on agriculture. Feng et al. proposed a deformable convolutional and expansive convolutional neural network for detection of ASR with 96.3% accuracy, but the detection target was too homogeneous to identify other types of damaged soybean seeds^22^.

**Fig. 1:**
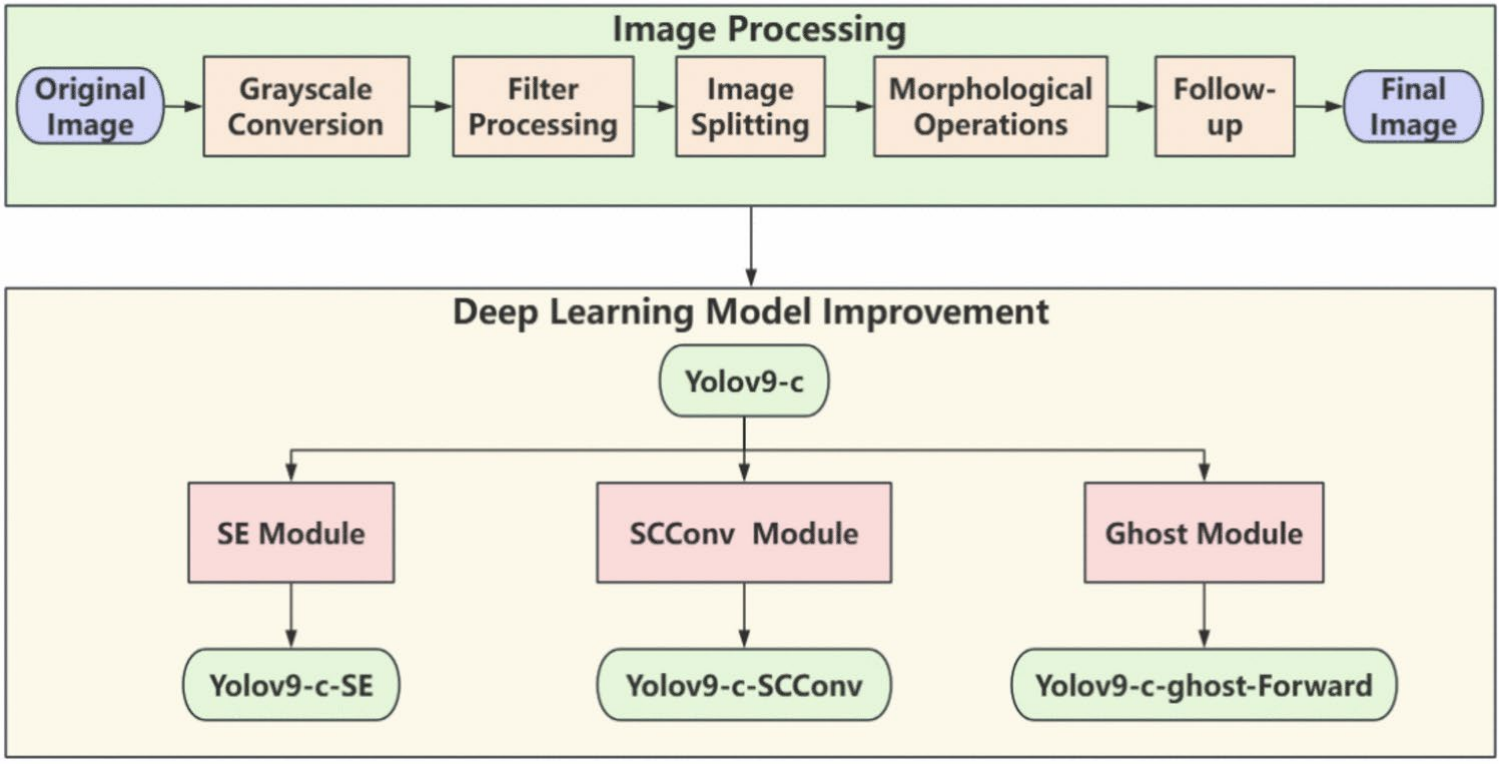
Modeling framework.

To address this gap, this study explores the use of deep learning models to detect soybean seed surface defects. Specifically, the YOLOv9 deep learning algorithm detects and classifies these defects automatically. To accomplish these objectives, a modeling framework has been developed, as shown in Fig. 1. It is detailed as follows:

(1) Data cleaning and preprocessing of soybean images. Grayscale conversion, filter processing, image segmentation, morphological operations, noise removal, and image merging were used to optimize the dataset, thus enhancing the model’s accuracy after training. (2) Various performances, such as detection accuracy, were improved by incorporating the SE attention mechanism in Yolov9-c and adding the SCConv self-calibrating convolution module and ghost module. This study used Yolov5s and Yolov9-c models to train the dataset according to the characteristics of soybean seed surface defects. On this basis, the SE attention mechanism was added to Yolov9-c, and the Yolov9-c-SE model was proposed. Then the SCConv self-calibrating convolution module from SCNet was added to Yolov9-c, introducing the Yolov9-c-SCConv model. This innovative approach enhances the receptive field of each convolutional layer by facilitating internal communication, thereby enriching the output features. Finally, the ghost module is introduced, and the Yolov9-c-ghost-Forward model is proposed. Finally, surface damage, speckles, and broken seeds in undesirable soybean seeds can be detected and recognized with improved detection accuracy. This study anticipates presenting an innovative methodology for evaluating soybean seed quality, accelerating advancements in agricultural science and technology, and furthering sustainable development practices in the agricultural sector.

## Materials and methods

### Selection of experimental samples

Aiming to detect various soybean seeds, this study selected four types of seeds from the dataset on the website: https://www.kaggle.com/datasets/warcoder/soyabean-seeds, namely intact seeds, damaged seeds, surface-damaged seeds, and spotted seeds, to cope with various real-world agricultural problems, thereby improving the generalization ability and robustness of the training model.

**Fig. 2:**
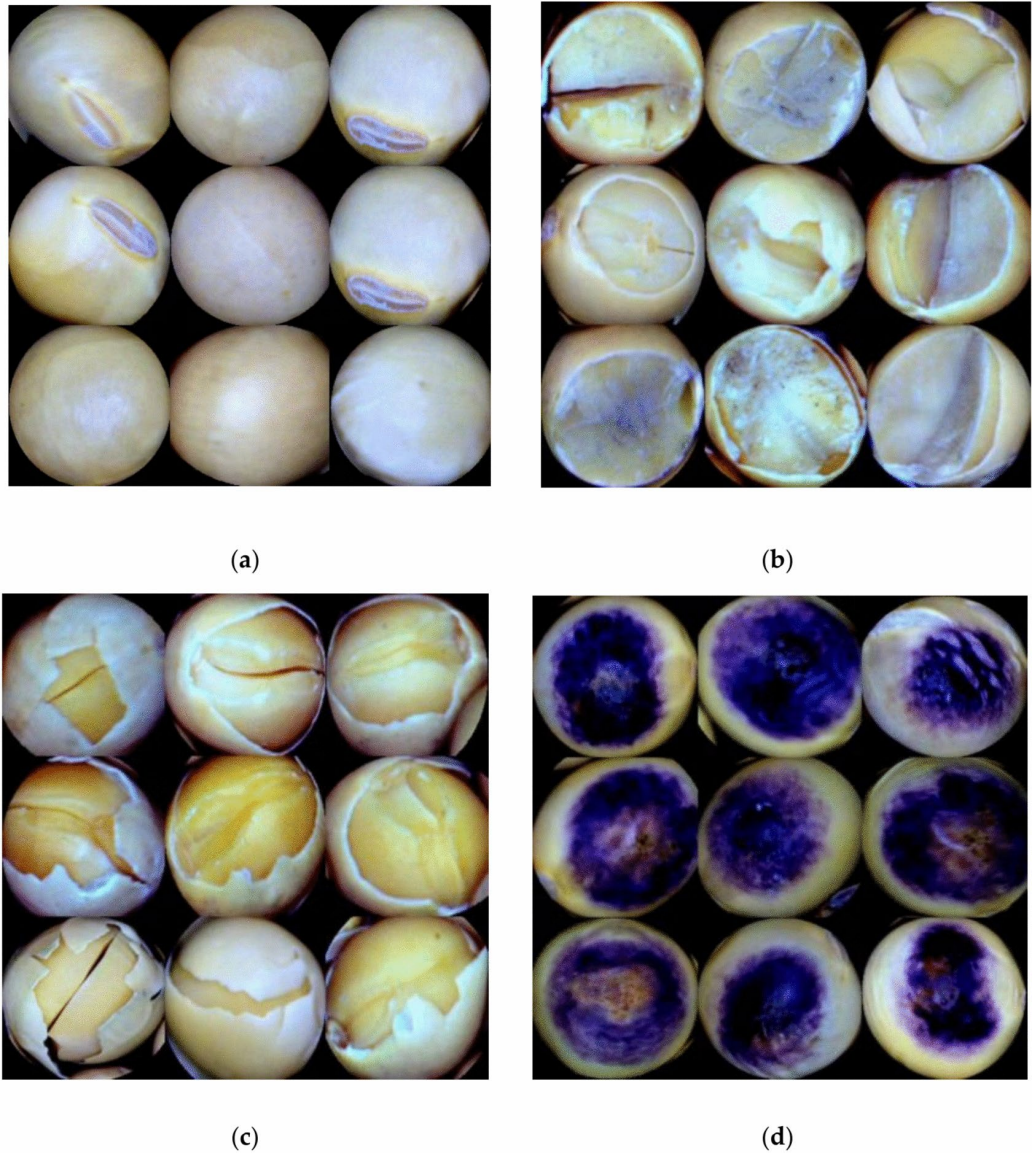
(**a**) Intact seed, (**b**) broken seed, (**c**) skin-damaged seed, (d) spotted seed.

Table 1 summarizes the experimental dataset comprising 4,388 samples, categorized into 1,201 intact seeds, 1,002 damaged seeds, 1,127 with surface damage, and 1,058 spotted seeds. The experimental samples consisted of two types: defective soybean seeds, including broken seeds, skin-damaged seeds, and spotted seeds, and non-defective soybean seeds, including only intact seeds. The collected images are shown in Fig. 2.

**Table 1:** Number of experimental data.

Types of soybean seeds	Quantities
Intact	1201
Broken	1002
Skin-damaged	1127
Spotted	1058
Total	4388

### Image processing

#### Grayscale conversion

Specifically, this study adopts a weighted average approach for grayscale conversion, as illustrated in Eq. (1). By using these weights, information loss can be effectively minimized while emphasizing the main features of the image.

1 $$Gray\left( {x,y} \right) = 0.299 \times R\left( {x,y} \right) + 0.587 \times G \left( {x,y} \right) + 0.114 \times B\left( {x,y} \right)$$

where $Gray$ denotes the grayscale, $R\left( {x,y} \right)$ the pixel value of the image’s red channel $\left( {x,y} \right)$,$G\left( {x,y} \right)$ the pixel value of the green channel $\left( {x,y} \right)$ , and $B\left( {x,y} \right)$ the pixel value of the blue color channel $\left( {x,y} \right)$.

#### Image Filtering

Filter processing removes noise or highlights features by applying filters to the image. This study proposes to use three filtering methods for data enhancement:

Median filtering, a nonlinear approach, which ensures that noise is reduced while maintaining the image’s structural integrity^23^. The specific calculation is shown in Eq. 2.

2 $$G\left( {x,y} \right) = median\left\{ {f\left( {i,j} \right)\left| {\left( {i,j} \right) \in W_{x,y} } \right.} \right\}$$

where $G\left( {x,y} \right)$ is the pixel value of the filtered image at the position $\left( {x,y} \right)$, $f\left( {i,j} \right)$ is the pixel value of the original image at the position $\left( {i,j} \right)$ within the $W_{x,y}$ window, and $W_{x,y}$ is the window centered on $\left( {x,y} \right)$.

Mean filtering can eliminate high-frequency disturbances, but may introduce a side effect of diminishing the sharpness or clarity of the image’s edges^24,25^. The specific calculation is shown in Eq. (3).

3 $$G(x,y) = \frac{1}{{k^{2} }}\sum\nolimits_{{(i,j) \in W_{x,y} }} {f(i,j)}$$

where $G\left( {x,y} \right)$ is the pixel value of the filtered image at position $\left( {x,y} \right)$, $f\left( {i,j} \right)$ is the pixel value of the original image at position $\left( {i,j} \right)$ within the window $W_{x,y}$, and *k* is the width and height of the window (assuming the window is square).

Gaussian filtering ensures that the image is smoothed while preserving important edge details^26^. The specific calculation is shown in Eq. (4).

4 $$G\left( {x,y} \right) = \sum\nolimits_{{\left( {i,j} \right) \in \mathop W\nolimits_{x,y} }} G \left( {i,j} \right) \cdot f\left( {i,j} \right)$$

where $G\left( {x,y} \right)$ the pixel value of the filtered image is at position, the pixel value of the original image is at position within the window, and the Gaussian weight is at position $\left( {i,j} \right)$ within the window.

#### Image segmentation

The Otsu algorithm is particularly advantageous as it maximizes the variance between classes, ensuring a clear distinction between foreground and background elements. Furthermore, the Otsu method streamlines the segmentation process by eliminating the need for manual threshold selection and enhancing efficiency and accuracy^27^. The specific calculation is shown in Eqs. (5) and (6).

5 $$\sigma_{inter}^{2} (t) = \sum\nolimits_{i = 0}^{t} {p(i)} \cdot \sum\nolimits_{i = t + 1}^{L - 1} {p(i)} \cdot \left[ {\sum\nolimits_{i = 0}^{t} {i \cdot p(i)} - \sum\nolimits_{i = t + 1}^{L - 1} {i \cdot p(i)} } \right]$$

6 $$threshold = \arg \max \;\sigma_{inter}^{2} (t)$$

where $\sum\nolimits_{i = 0}^{t} {p\left( i \right)}$ denotes the probability of the background, $\sum\nolimits_{i = t + 1}^{L - 1} {p\left( i \right)}$ the probability of the foreground, the mean gray value of the background, and the mean gray value of the foreground.

#### Morphological operations

Morphological operations primarily analyze and manipulate image shapes by identifying and altering their constituent structural elements.

### Labeling the dataset

There are a total of five elements in the file.The first element identifies the target category, while the subsequent four elements detail the position and dimensions of the bounding box.

### Yolo series models

Yolov5s, as a lighter version of the Yolov5 series, can do so while maintaining accuracy designed for efficient real-time target detection. The model’s architecture is divided into three parts: Backbone, Neck, and Head, each containing multiple modules that build a robust detection network.

In the Yolov5 family of models, Yolov5s is a superior choice due to its compact size, exceptional detection efficiency, and commendable accuracy. Incorporating the CSP (Cross Stage Partial) module into Yolov5 further enhances its capabilities. Specifically, its backbone network comprises a meticulous architecture of ten distinct modules, including the CBS, C3, and SPPF modules, each contributing to the model’s overall performance, as detailed in Table 2. This intricate combination of modules keeps the model lightweight and ensures high-quality detections with great precision.

**Table 2:** The backbone network structure of Yolov5s.

Serial number	1	2	3	4	5	6	7	8	9	10
Module Name	CBS	CBS	C3	CBS	C3	CBS	C3	CBS	C3	SPPF
Output size	320 × 320 × 32	160 × 160 × 64	160 × 160 × 64	80 × 80 × 128	80 × 80 × 128	40 × 40 × 256	40 × 40 × 256	20 × 20 × 512	20 × 20 × 512	20 × 20 × 512

#### PGI collection

Yolov9 incorporates the PGI (Programmable Gradient Information) framework, structured around three key elements: a primary branch, an auxiliary reversible branch, and multilevel auxiliary information. Each component plays a vital role in the framework. PGI is used only in the main branch during inference, so there is no additional inference cost for the other branches. As the neural network deepens, it causes the information bottleneck problem, which results in the loss function being unable to generate reliable gradients. At the same time, the auxiliary reversible branch handles the problems caused by the deepening of the neural network. Deep supervision tends to introduce the problem of error accumulation when inference is performed using architectures with multiple predictive branches and lightweight models, which can be addressed by various levels of auxiliary information.

This helps generate reliable gradients by aiding reversible branching and ensuring that deep features retain the key features needed to perform the target task. This improvement addresses the tendency of deep neural networks to lose information during feedforward.

#### GELAN architecture

**Fig. 3:**
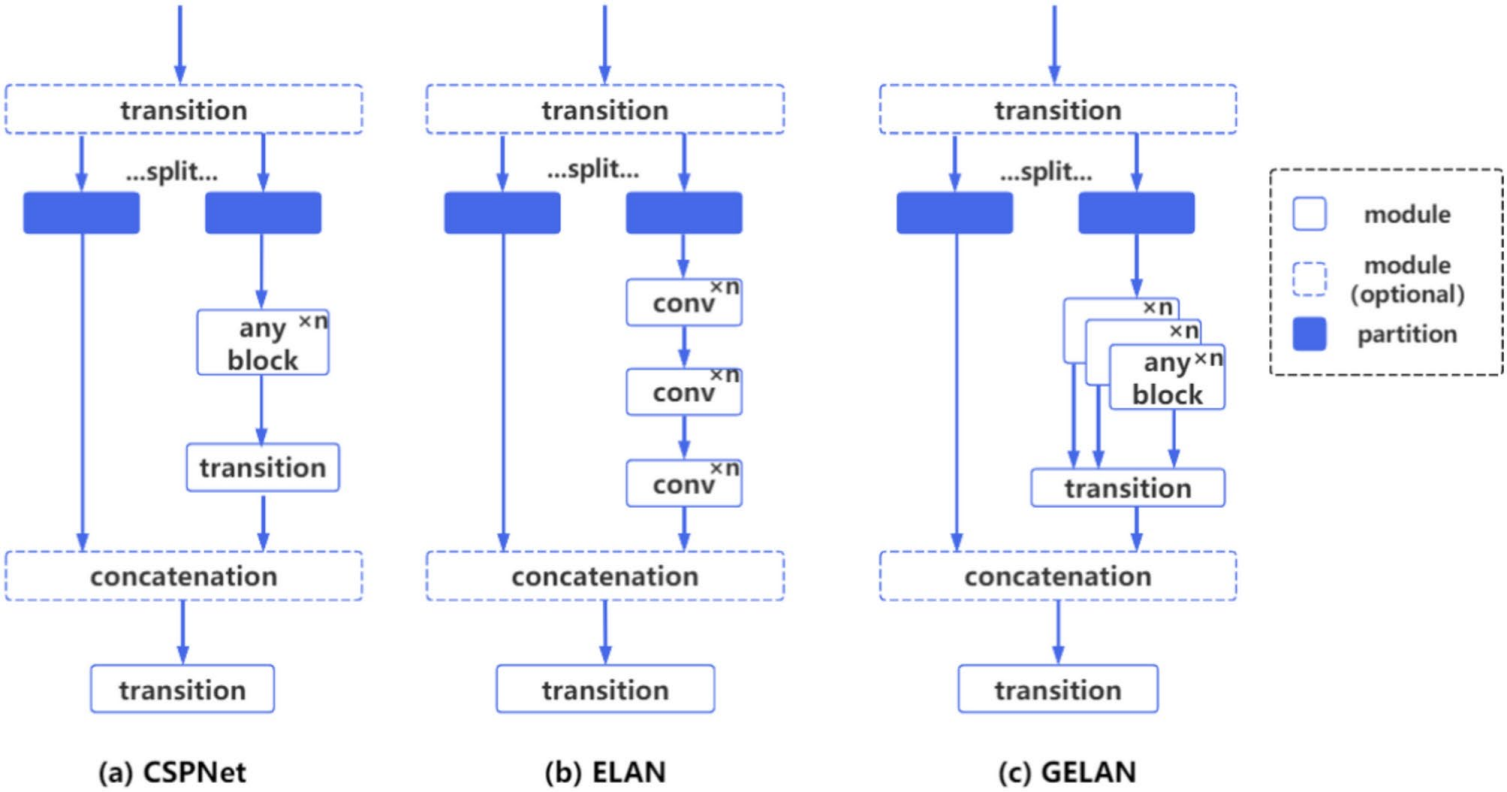
Comparison of CSPNet, ELAN, and GELAN network structures.

Unlike the CSPNet architecture used by Yolov5 and the ELAN architecture used by Yolov7, Yolov9 generalizes the capabilities of ELAN by combining these two neural network architectures designed using gradient path planning, improving from the previous stacking using only convolutional layers to the ability to use any computational block as a base Module, and developing this generalized effective layer aggregation network (GELAN). The illustration in Fig. 3 showcases the network architecture and its comparative features. This design balances the parameters’ count, computational intricacy, accuracy, and inference velocity while empowering users to tailor the computational blocks to diverse device requirements, amplifying the model’s adaptability and efficiency. Its backbone network structure is shown in Table 3.

**Table 3:** Yolov9 backbone network structure.

Serial number	1	2	3	4	5	6	7	8	9
Module name	Conv	Conv	RepNSCPELAN4	Conv	RepNSCPELAN4	Conv	RepNSCPELAN4	Conv	RepNSCPELAN4

#### RepNSCPELAN4 module

This module mainly integrates the CSPNet Block module of Yolov5, the Rep module of Yolov6, and the ELAN module of Yolov7. It inherits CSPNet’s cross-stage feature fusion, Rep’s lightweight design, and ELAN’s residual linkage, ensuring both lightweightness and robust inference & feature extraction. This blend enables Yolov9 to process image data proficiently, enhancing target detection and image classification accuracy and efficiency.

The Yolov9-c model extends Yolov9 by integrating the Adown convolutional block specifically for downsampling in object detection tasks. This innovation enables the model to extract high-level image features effectively while minimizing computational demands, achieving efficient processing without compromising model performance.

Its lightweight and high-performance design gives the model a more significant advantage when deployed on mobile and applied to agricultural breeding screening. Its backbone network structure is shown in Table 4:

**Table 4:** Yolov9-c backbone network structure.

Serial number	1	2	3	4	5	6	7	8	9
Module name	Conv	Conv	RepNSCPELAN4	Adown	RepNSCPELAN4	Adown	RepNSCPELAN4	Adown	RepNSCPELAN4

### Improvement of modules

#### SE attention mechanism

The SE (Squeeze-and-Excitation) attention mechanism enhances CNN performance by adaptively recalibrating feature channels^28^. It can adaptively assign weights to different channels of the feature map to enhance the extraction of key features. Soybean seed defects are usually manifested as subtle texture changes, surface depressions or colour anomalies, etc. These features are more prominent in certain channels, while the SE mechanism can focus attention on the channels containing defective features, suppress irrelevant information, and improve the sensitivity to the defective regions and the detection accuracy by means of global information aggregation and channel-level weight adjustment. Integrating the SE mechanism into YOLOv9, especially in the feature extraction stage, can significantly enhance the model’s characterisation ability and enable it to distinguish defective seeds from healthy ones more accurately.

**Fig. 4:**
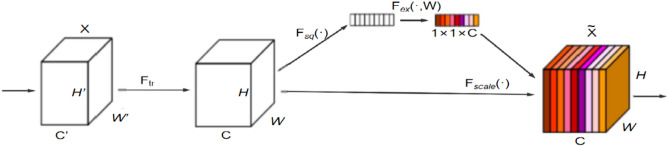
The Structure of the SE attention mechanism.

The SE module works in three main steps: compression, excitation, and scaling (as shown in Fig. 4). During compression, each channel’s spatial features are condensed into a single scalar using global average pooling (as in Eq. 7). In the excitation process, weights for each channel are produced via two FC layers and nonlinear activations (ReLU, Sigmoid). First, *Z* it is mapped to a low-dimensional space through a fully connected layer, followed by mapping it back to the original dimension through a fully connected layer (as shown in Eq. 8). Finally, the generated weights *s* are multiplied with the original feature map on an element-by-element basis by channel to recalibrate the importance of the channels (as shown in Eq. 9).

7 $$z_{c} = \frac{1}{H \times W}\sum\limits_{i = 1}^{H} {\sum\limits_{j = 1}^{W} {X{}_{c}\left( {i,j} \right)} }$$

Suppose the input feature map is $X \in R^{H \times W \times C}$ where *H* the height *W* is the width and *C* the number of channels. The output of global average pooling is $Z \in R^{C}$.

8 $$s = \sigma \left( {W_{2} \cdot \delta \left( {W_{1} \cdot Z} \right)} \right)$$

where $W_{1} \in R^{{\frac{C}{r} \times C}}$ and $W_{2} \in R^{{C \times \frac{C}{r}}}$ are the learnable weight matrices $\delta$ denote the ReLU activation function, $\sigma$ the Sigmoid activation function, and *r* the scaling factor.

9 $$\tilde{X}_{c} = s_{c} \cdot X_{c}$$

where $\tilde{X}_{c}$ is the output of the *c* th channel, and *s*_*c*_ is the weight of the *c*th channel.

#### SCConv

The SCConv (Self-Calibrated Convolution) module is an improved convolution operation designed to enhance the representation of local and global features in Convolutional Neural Networks (CNNs)^29^.

**Fig. 5:**
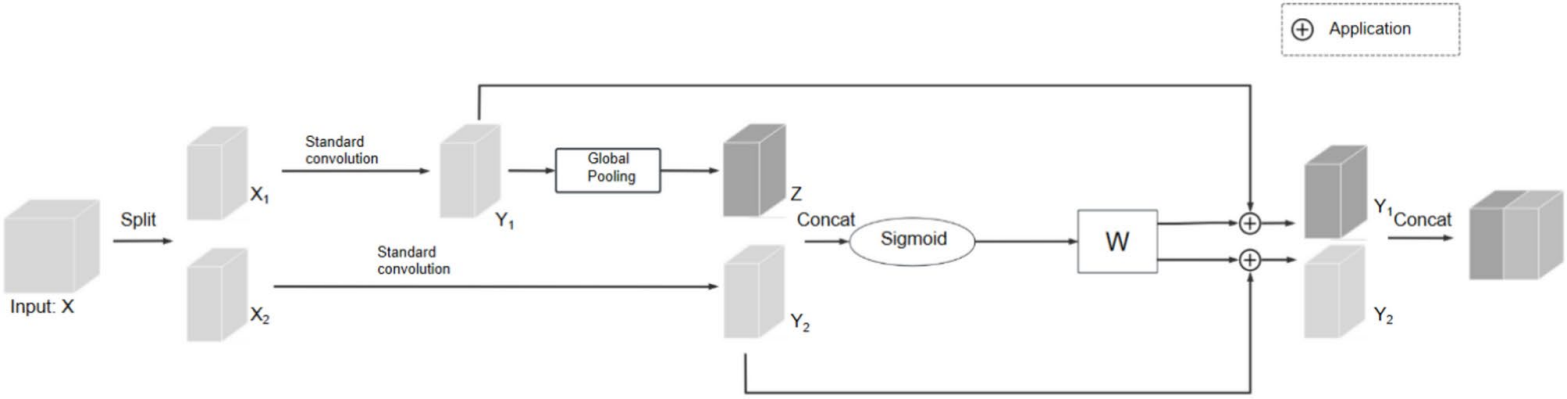
Workflow of the self-calibrating convolution module.

The design of SCConv consists of two key steps: generating calibration feature maps and fusing calibration feature maps. It consists of three main parts: standard convolution, feature transformation, and feature fusion, and the specific flow is shown in Fig. 5.

The SCConv module seamlessly integrates global and local contextual information, enriching feature representation. This enhancement notably boosts performance in image classification and object detection tasks without substantial computational overhead. Meanwhile, the SCConv module enhances the network’s ability to represent fine-grained features by introducing a feature self-calibration mechanism. The SCConv module introduces a parallel branch in the convolution process, extracting information from both the fine-grained and global context levels, respectively, and adaptively adjusting the response of the feature maps through the fusion operation. This mechanism can effectively reduce texture interference, while highlighting the tiny texture changes and colour anomalies commonly found in soybean seed defects, thus improving the model’s ability to identify defective regions, which can significantly enhance the robustness and sensitivity of the feature extraction stage.

#### Ghost module

**Fig. 6:**
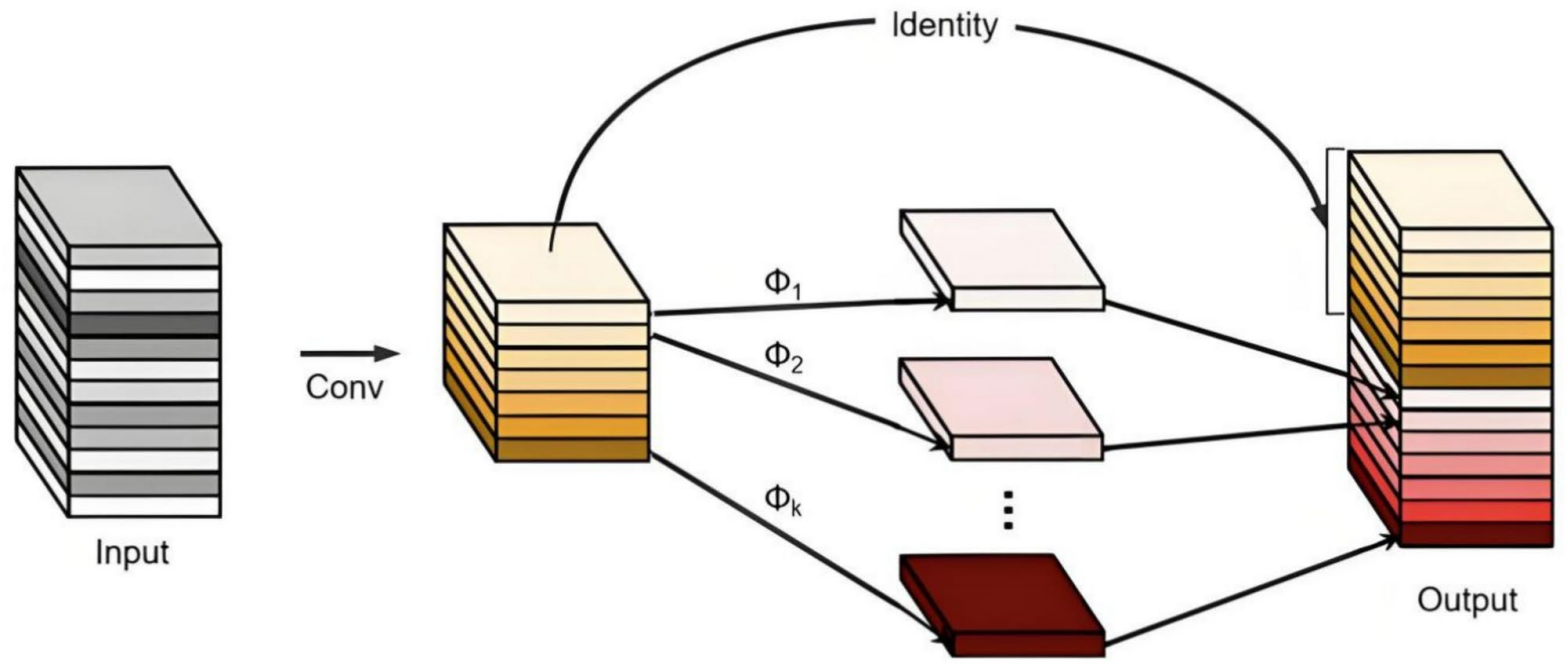
The Structure of Ghost Module.

The ghost module is an efficient structural module for convolutional neural networks^30^, an innovative design for convolutional neural networks, and its core idea is to generate more feature maps by low-cost linear transformations. The goal is to enhance the efficiency of convolutional neural networks by minimizing computation and parameter count, as depicted in Fig. 6. This optimized structure achieves this objective.

The ghost module generates feature maps in two steps: primary feature map generation and redundant feature map generation. First, a few master feature maps are generated using standard convolution operations. Then, a series of simple linear transformations (e.g., point-by-point convolution, depth-separable convolution) are applied to each master feature map to generate redundant feature maps. The result of these linear transformations is called the Ghost feature map.

The Ghost module significantly reduces the computational effort and number of parameters of the model by generating more efficient feature representations, while maintaining the ability to extract key features.The core of the Ghost module is the use of a small number of standard convolutions to generate the base features, and then generating redundant ‘pseudo-features’ through linear transformations or simple manipulations to This reduces computational complexity. Soybean seed defects are often characterized by subtle surface texture variations and colour differences, which require high-resolution feature extraction, and the Ghost module reduces redundant computation while retaining and highlighting these subtle features to improve detection accuracy. Furthermore, the ghost module’s versatility allows seamless integration into diverse convolutional neural network architectures.

### Indicators for model evaluation

In this research, the upgraded model’s proficiency in identifying surface defects in soybean seeds will be evaluated using three pivotal metrics, namely:

In assessing classifier performance, precision, a widely employed metric, signifies the proportion of accurately predicted samples among all predictions made by the model. Mathematically, it is expressed as the ratio of correctly predicted samples to the total number of samples, as shown in Eq. (10).

10 $$precision = \frac{TP}{{TP + FP}}$$

where *TP* (True Positive) is the number of samples where the model predicted and had positive cases, and *FP* (False Positive) is the number of samples where the model predicted positive cases but had negative cases (also known as false positives).

Recall is the proportion of all positive samples correctly predicted as positive, as defined in Eq. (11).

11 $$recall = \frac{TP}{{TP + FN}}$$

where *TP* is the number of samples where the model predicted and had positive cases, *FN* (False Negatives) is the number of samples where the model predicted negative cases and had positive cases (also known as underreporting).

The mAP0.5 is the average accuracy when the IoU threshold is 0.5, which is calculated as shown in Eq. (12).

12 $$mAP0.5 = \frac{{TP_{1} + TP_{2} + \cdots + TP_{n} }}{{TP_{1} + TP_{2} + \cdots + TP_{n} + FP_{1} + FP_{2} + \cdots + FP_{n} }}$$

where *TP*_*i*_ denotes the number of correctly predicted samples in the category *i* and *FP*_*i*_ the number of incorrectly predicted samples in the category *i*.

The P-R Curve (Precision-Recall Curve) graph has recall as the horizontal axis, and precision as the vertical axis, and these two are calculated as shown in Eqs. (11) and (10).

The ROC curve (Receiver Operating Characteristic Curve) is plotted with the true case rate (*TPR*) as the vertical axis, and the false positive case rate (*FPR*) as the horizontal axis, and the specific calculation method is shown in Eqs. (13) and (14).

13 $$TPR = \frac{TP}{{TP + FN}}$$

14 $$FPR = \frac{FP}{{TN + FP}}$$

where *TP* is the number of samples in which the model predicted and actualized positive cases, *FP* is the number of samples in which the model predicted positive cases and actualized negative cases, *TN* is the number of samples in which the model predicted and actualized negative cases, and *FN* is the number of samples in which the model predicted negative cases and actualized positive cases.

## Results

### Model parameter selection and training

For this research, the computational setup comprised an Intel i7-12700F CPU clocked at 4.9 GHz, complemented by 32 GB of RAM and an NVIDIA GeForce RTX 4090 GPU with 24 GB of memory. The operating system was Windows 11, and Python 3.8 was the programming language. The deep learning frameworks employed were PyTorch 2.3 and CUDA 11.7. Stochastic Gradient Descent (SGD) was selected as the optimization algorithm, with a batch size 16. The learning rate was initialized at 0.01, and a learning rate factor of 0.01 was also set. After 200 training epochs, the objective was to develop a system capable of facilitating agricultural breeding screening.

### Image preprocessing

This paper uses image preprocessing methods, including grayscale conversion, filtering processing, threshold segmentation, binarization processing, and morphological operations to reduce the impact of noise caused by variations in light and brightness, as well as interference with the training model.

#### Grayscale conversion

**Fig. 7:**
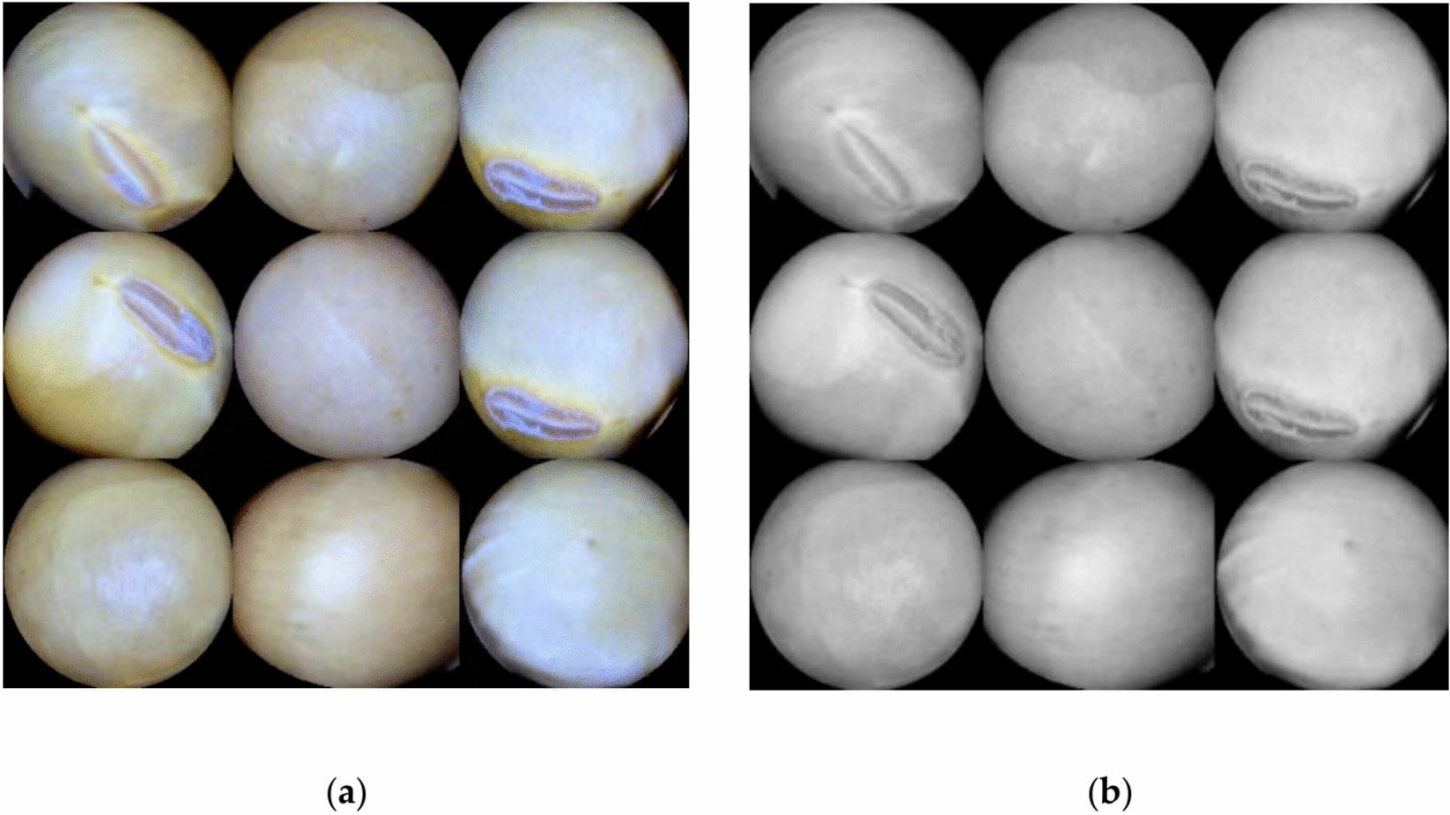
Comparison of image before and after grayscale processing.

As evident in Fig. 7, the soybean image’s clarity is hindered by noise in the grayscale representation, likely caused by factors such as lighting angles during image capture. This noise leads to poor contrast, necessitating image enhancement. Studies suggest that heightened image contrast correlates positively with recognition accuracy when trained with deep learning algorithms^31^. Hence, minimizing external image distortions and enhancing contrast is crucial for this research to enhance recognition accuracy. Among various image enhancement techniques, filtering methods—notably median, mean, and Gaussian filtering—prove effective in noise reduction and overall image quality improvement.

#### Filter processing

For this study, image filtering was applied using three distinct filters: median, mean, and Gaussian. As shown in Table 5, several sets of experiments were conducted by adjusting the kernel parameters and the signal-to-noise ratio of each combination was calculated by the code for quantitative evaluation of the filtering results.

**Table 5:** Table of experimental conditions for image filtering.

Groups	Filter type	Kernel size	SNR
1	Median filter	3 × 3	1.940
2	Median filter	5 × 5	1.945
3	Median filter	7 × 7	1.941
4	Mean value filter	3 × 3	1.938
5	Mean value filter	5 × 5	1.939
6	Mean value filter	7 × 7	1.944
7	Gaussian filter	3 × 3	1.940
8	Gaussian filter	5 × 5	1.951
9	Gaussian filter	7 × 7	1.944

**Fig. 8:**
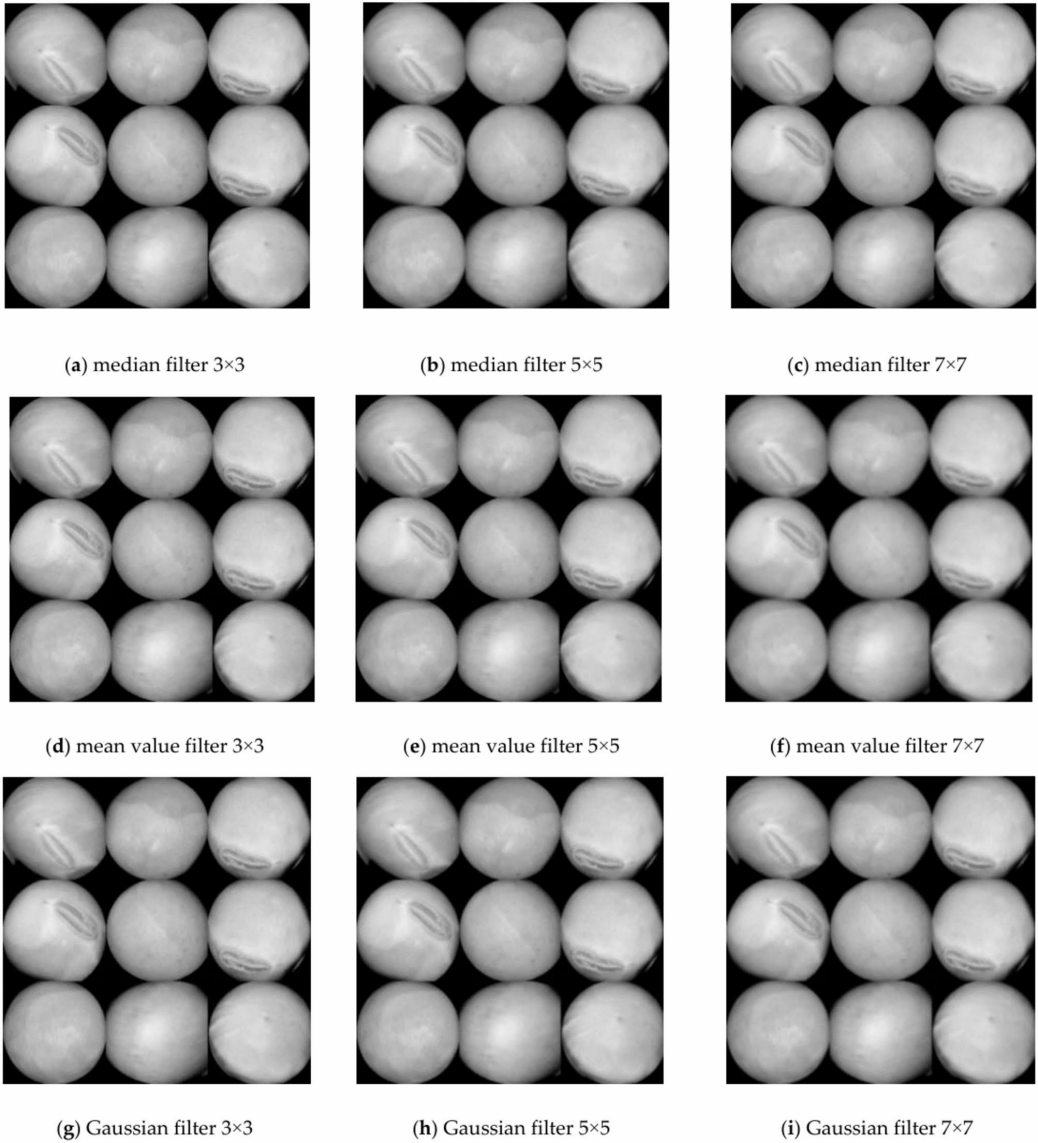
The image filtering results.

In Fig. 8a to (i), The image filtering results correspond to the experimental conditions of groups 1 to 9 in Table 5.

As we all know, the signal-to-noise ratio(SNR) is a measure of the ratio of the filtered image signal to the noise, and the higher the SNR, the better the image quality.Based on the data in Table 5, it can be learnt that the quality of the image is highest when a Gaussian filter is used and filtered with a 5 × 5 convolution kernel and hence this combination is used for the pre-processing of the image.

#### Image segmentation

**Fig. 9:**
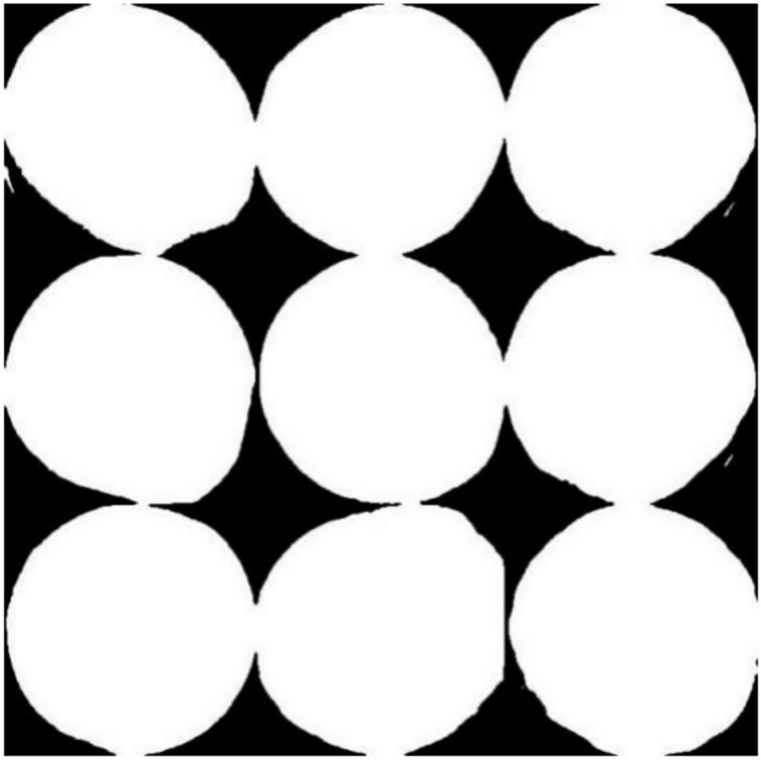
The result after segmentation using the OTSU algorithm.

Upon processing, the optimal threshold was attained. The obtained image is depicted in Fig. 9; from the image, the segmented boundary is obvious, and the distinction between the foreground and background is high, but there is still hole noise and point noise in the image.

#### Morphological operations

After applying grayscale conversion, Gaussian filtering, and threshold segmentation, the image’s quality and detail were significantly improved compared to its original state. However, despite these enhancements, a minimal amount of noise remained. This study incorporated morphological operations into the image processing pipeline to refine image quality and data integrity.

**Fig. 10:**
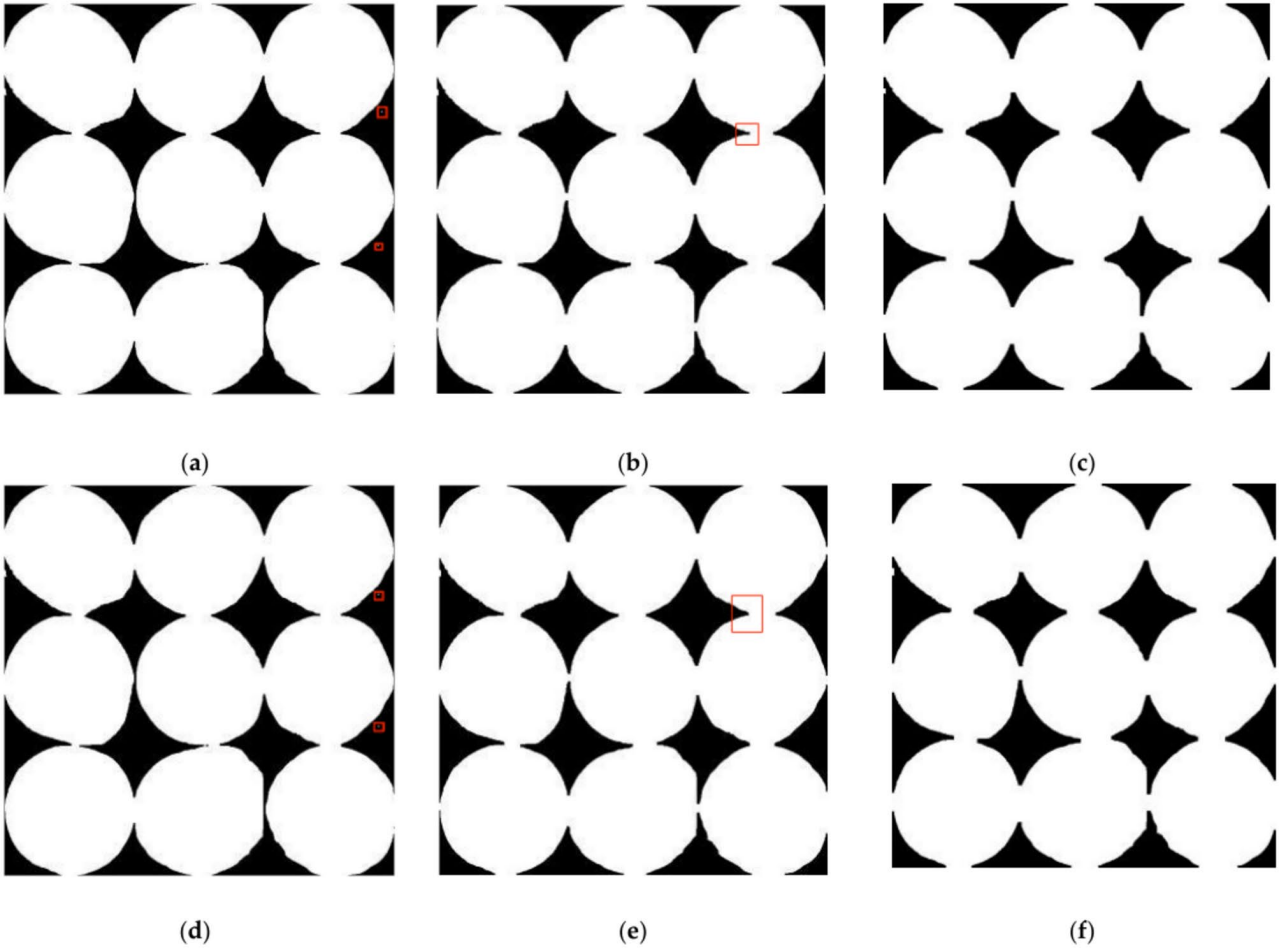
The morphological operations results.

After threshold segmentation on the image with varying kernel sizes (3 × 3, 5 × 5, and 7 × 7), opening and closing operations were sequentially applied in alternating orders, resulting in six experimental sets outlined in Table 6. The outcomes of these morphological operations, which align with the experimental conditions specified in groups 1 to 6 of Table 3, are presented in Fig. 10, with (a) through (f) depicting the respective results.

**Table 6:** Table of experimental conditions for morphological operations.

Batch number	Kernel size	Operating sequence
1	3 × 3	Open and closed operations
2	5 × 5	Open and closed operations
3	7 × 7	Open and closed operations
4	3 × 3	Closed operations, open operations
5	5 × 5	Closed operations, open operations
6	7 × 7	Closed operations, open operations

The experimental outcomes reveal that a 5 × 5 kernel matrix, when applied to slide over the input image, achieves a balance between noise reduction and preservation of image boundaries. It efficiently eliminates hole and point noise while minimally altering the original boundary characteristics of the image. Conversely, a 3 × 3 matrix, though gentle on the geometric features of soybean seeds, exhibits less pronounced noise removal than larger kernels. On the other hand, a 7 × 7 kernel excels in removing noise from soybean grains. Still, it overly smooths their geometric features, causing blurring and the filling of incomplete boundaries in the original image.

Hence, this study concludes that utilizing 5 × 5 matrices as kernels ensures minimal disruption to the geometric features of the image’s targets while effectively diminishing hole and point noise on soybean seeds. In addition, employing three distinct matrix sizes was effective when performing open and close operations on the soybean seed image in this experiment. The sequence of performing open followed by close operations worked significantly better than the sequence of performing.performing close and open operations.

#### Hole filling operations

**Fig. 11:**
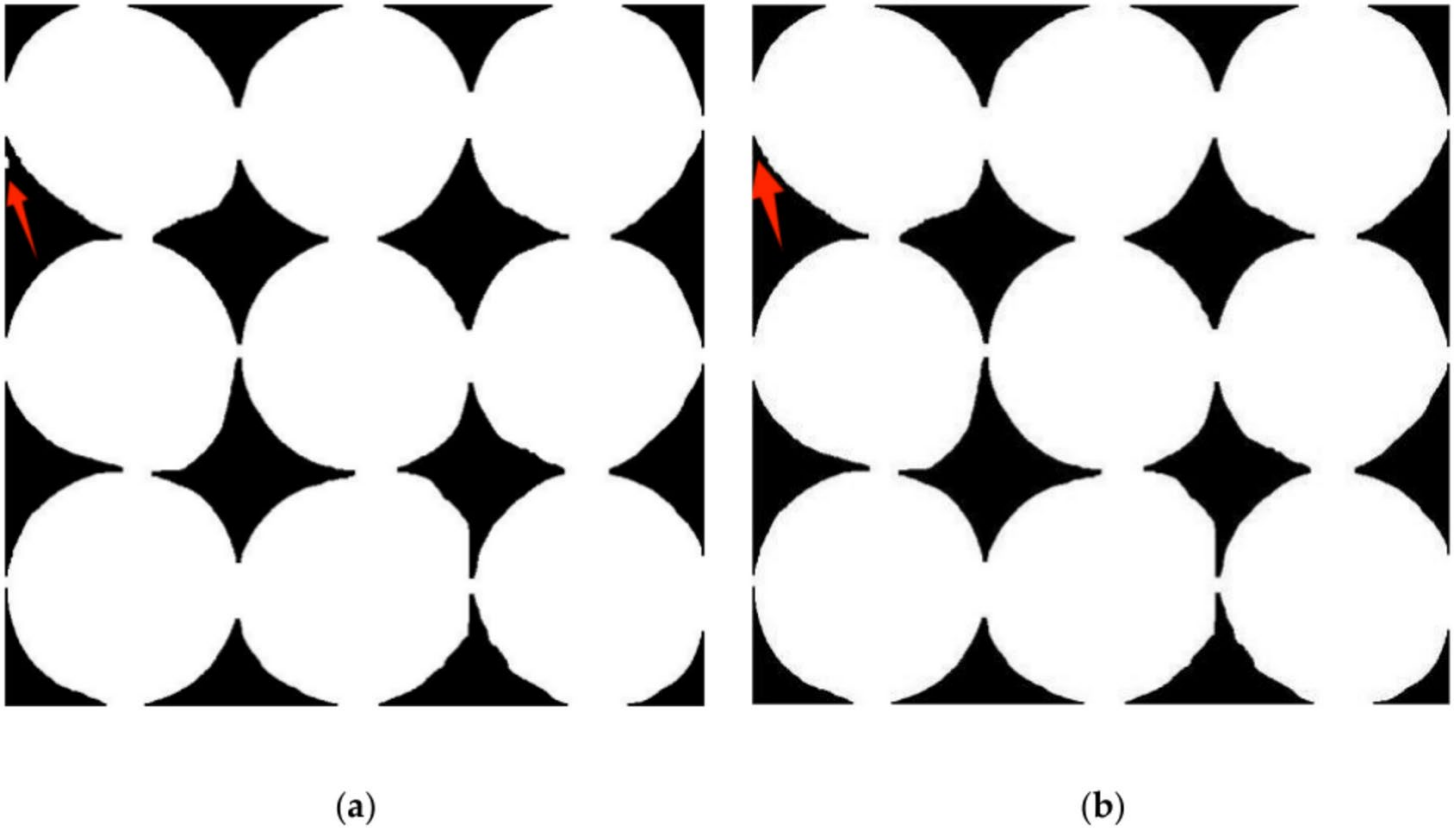
Comparison between hole filling operation and small area clear operation before (**a**) and after (**b**).

**Fig. 12:**
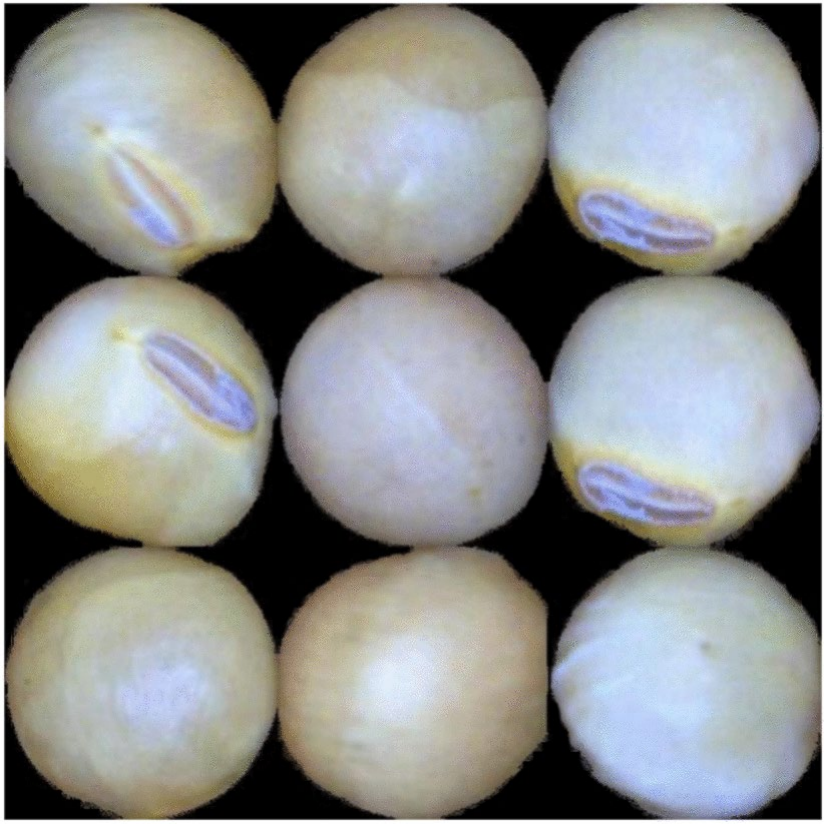
Final image.

Following the application of open and closed operations, a significant reduction in background noise is observed, yet point noise persists within the binary image of the soybean seeds. To prevent these noises from impacting subsequent model training, this study uses a hole-filling operation to fill the hole regions in the image. After the closing operation, noise in the target kernel can also be removed. After hole filling, a small area removal process is applied to the binary soybean kernel image, resulting in the refined image presented in Fig. 11. This morphological processing enhances the visibility of the soybean kernel’s shape and geometric properties while eliminating noise. The refined binary image is then fused with the source image through binary operations and bit manipulation, producing an output where the soybean grains retain their appearance. At the same time, the background is transformed into a solid black, as depicted in Fig. 12. With its clear depiction of soybean seeds and pronounced features, this processed image is well-suited for classification training within deep-learning convolutional neural networks.

### Labeling of data sets

**Fig. 13:**
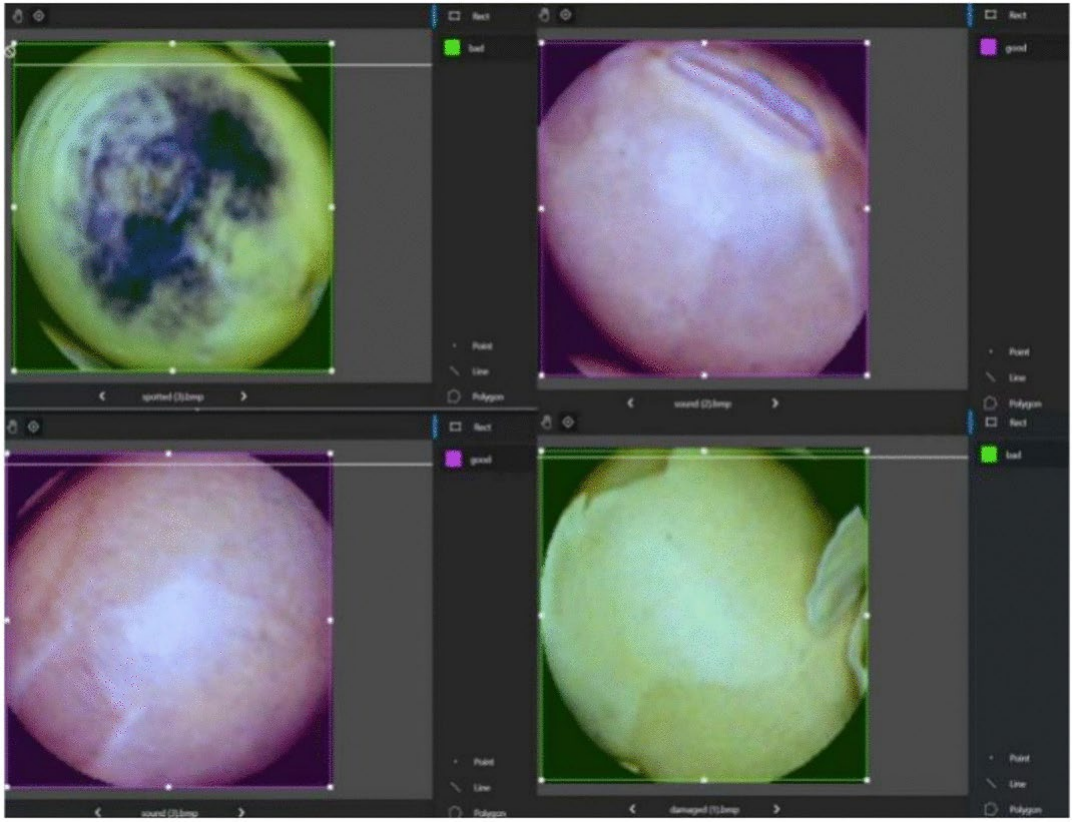
Labeling process of the dataset.

There are several ways to annotate an image; one of the most common methods is using an annotation tool. In this study, the tool https://www.makesense.ai/ was used for the annotation process illustrated in Fig. 13.

The annotation phase involved categorizing soybean seeds into “good” for those with superior appearance and “bad” for those exhibiting defective surfaces. These labels were utilized to assess the model’s performance. The comprehensive dataset for this study comprises two primary components: “images” and "labels," both of which are subdivided into “training” and “testing” folders. Specifically, the dataset was split into training and test sets, with a 4:1 ratio, totaling 4388 images. This distribution facilitates model training and evaluation.

### The improvement based on the Yolov9 model

#### Yolov9-C model

**Fig. 14:**
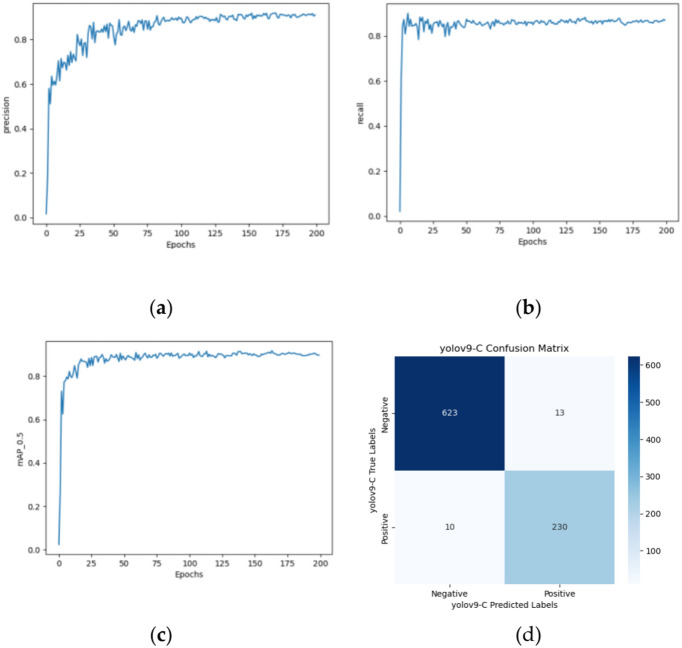
Yolov9-c's (**a**) precision growth curve, (**b**) recall growth curve, (**c**) mAP0.5 growth curve, and confusion matrixs.

After image enhancement, higher quality and more distinctive data were obtained, and the Yolov9-c model was trained with a network depth of 962 layers and 51,001,900 parameters. The obtained training results are shown in Fig. 14a–c.

#### Introduction of SE attention mechanisms

**Fig. 15:**
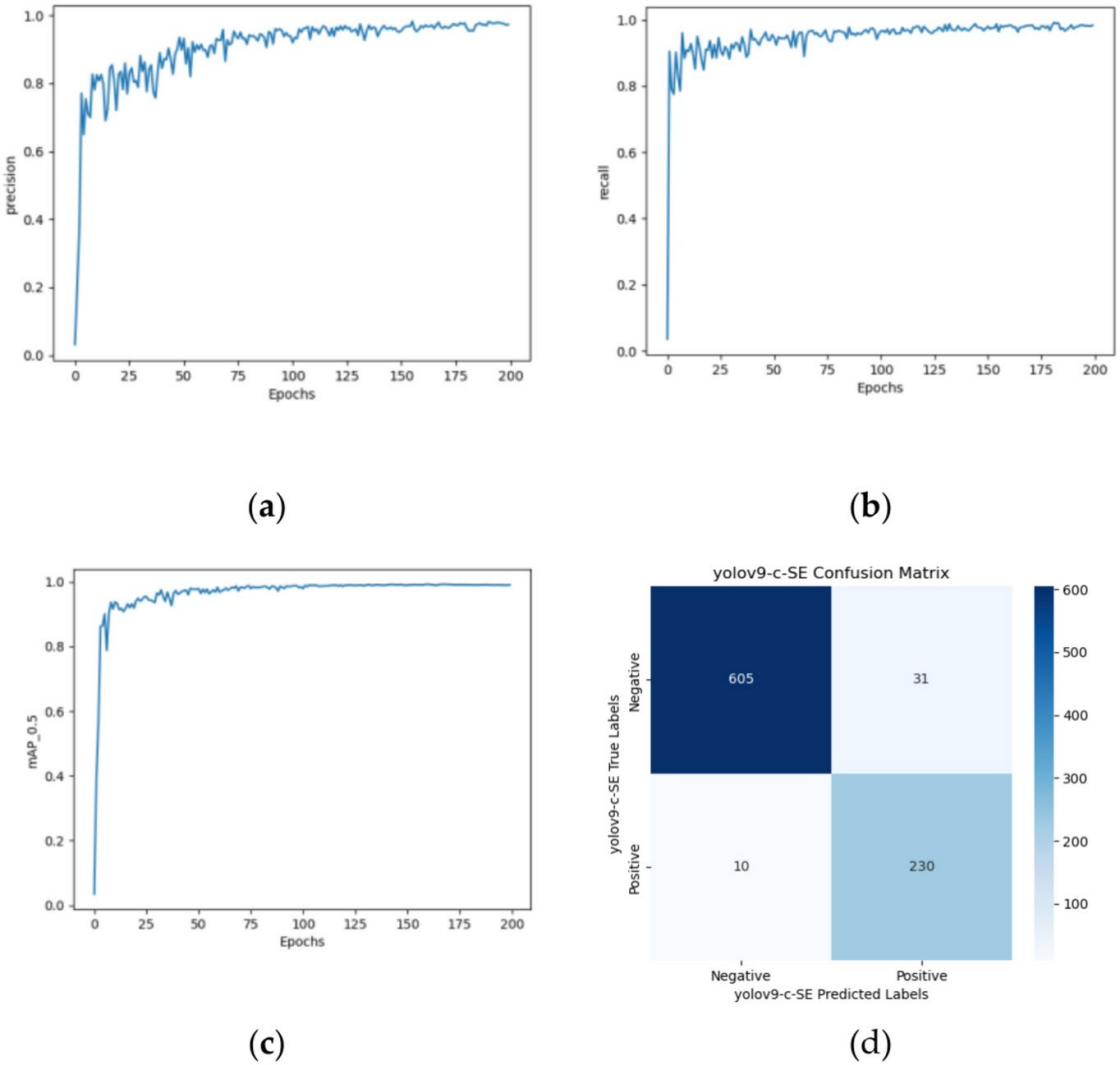
Yolov9-c-SE’s (**a**) precision growth curve, (**b**) recall growth curve, (**c**) mAP0.5 growth curve, and confusion matrixs.

To enhance the model’s performance, the SE (Squeeze-and-Excitation) module is initially incorporated into the "common.py" script and subsequently registered in the "yolo.py" file. A duplicate Yolov9-c configuration file is created and named "Yolov9-c-SE. yaml". By editing the contents of this configuration file, the SE attention mechanism is seamlessly integrated into the Yolov9-c architecture. The revised model boasts 969 layers and comprises 51,034,688 parameters, representing a modest 0.7% increase in layers and a mere 0.06% augmentation in parameters compared to the original Yolov9-c. Figure 15a–c depict the resultant training outcomes.

#### Introducing the SCConv module

**Fig. 16:**
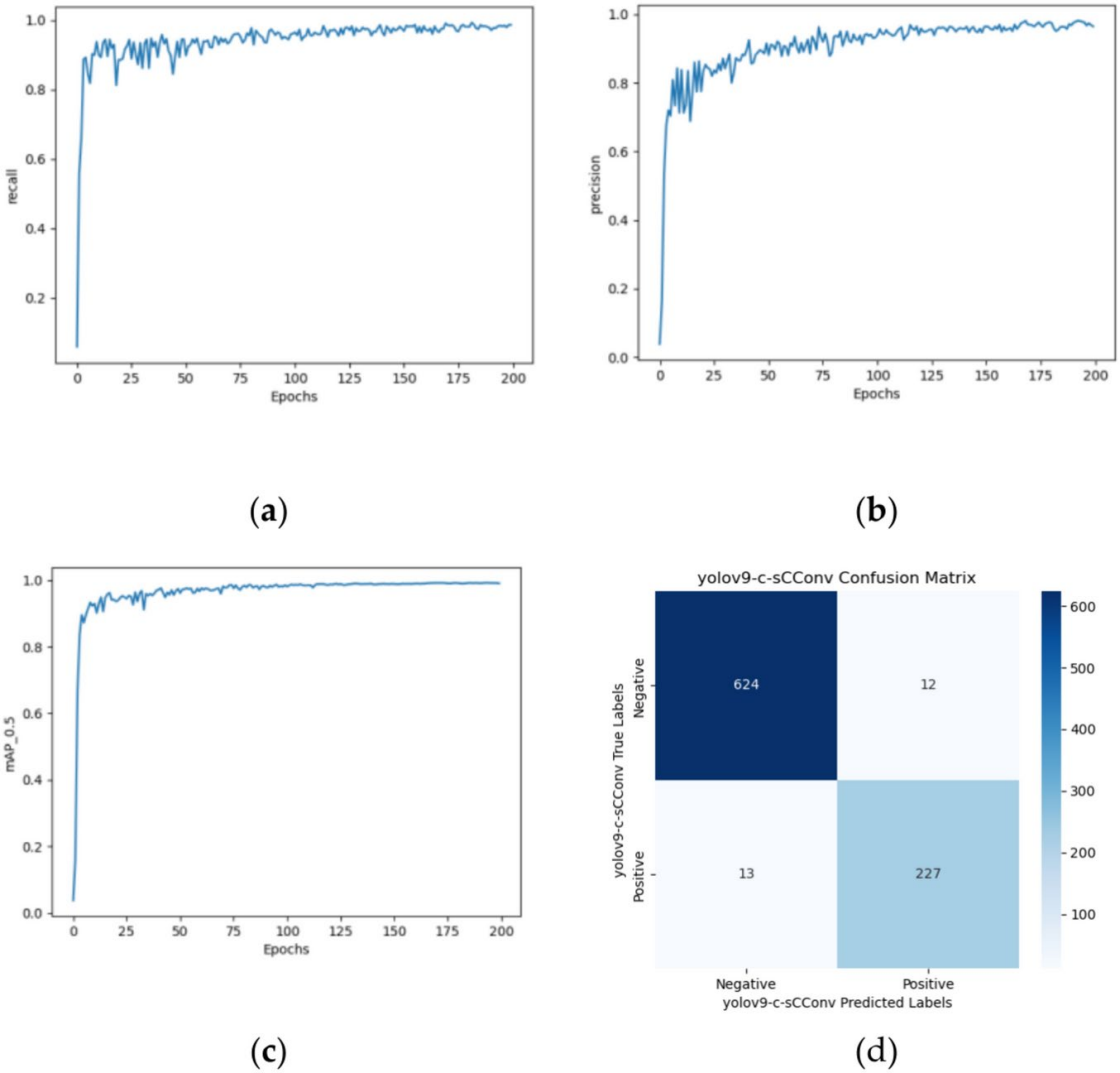
Yolov9-c-SCConv’s (**a**) precision growth curve, (**b**) recall growth curve, (**c**) mAP0.5 growth curve, and confusion matrixs.

To replace some of the RepNSCPELAN4 modules in Yolov9-c with the SCConv module in SCNet, you need to first add the SCConv module to the "common.py" file and register it to the "yolo.py and register it in the "yolo.py" file. Next, create a duplicate of the Yolov9-c config file and name it "Yolov9-c-SCConv.yaml", and finally, add the SCConv module to Yolov9-c by modifying the configuration file’s contents. The modified model has 910 layers and 55,225,388 parameters. Compared with Yolov9-c-SE, although the number of model layers decreased by about 7%, the number of parameters increased by 5%. Figure 16a–c shows the training results obtained.

#### Introducing the Ghost Conv module

**Fig. 17:**
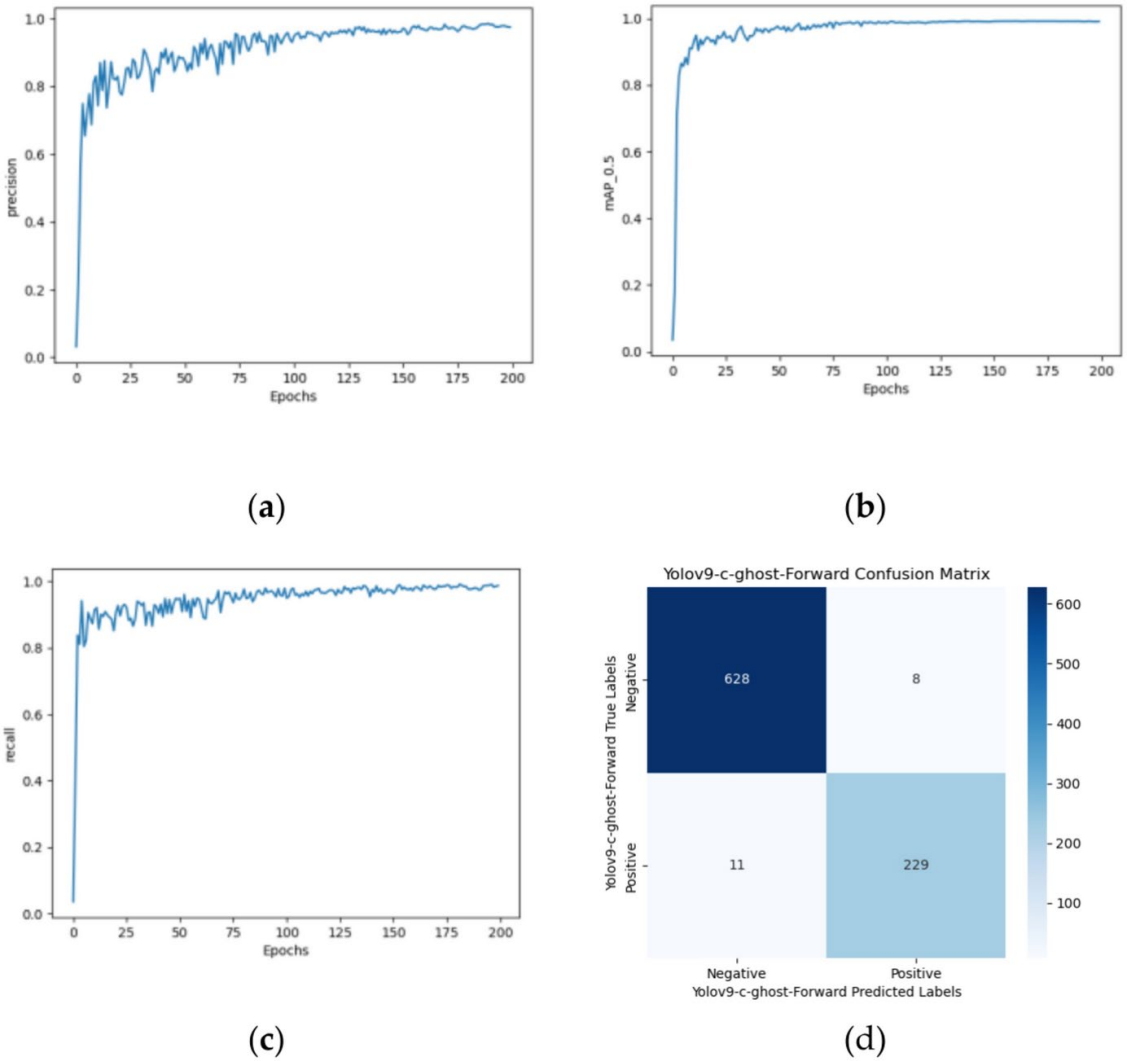
Yolov9-c-ghost-forward’s (**a**) precision growth curve, (**b**) recall growth curve, (**c**) mAP0.5 growth curve, and confusion matrixs.

To add the Ghost Conv module to Yolov9-c, first add the Ghost Conv module to the "common.py" file and register it in the "yolo.py" file. Next, create a duplicate of the Yolov9-c config file and name it "Yolov9-c-ghost-forward. yaml", and finally, add the Ghost Conv module to Yolov9-c by modifying the configuration file’s contents. The modified model has 969 layers and 52,188,972 parameters. Compared with Yolov9-c-SE, although the quantity of model layers remains unchanged, the quantity of parameters increases by 2%. The training results are shown in Fig. 17a–c.

## Discussion

**Fig. 18:**
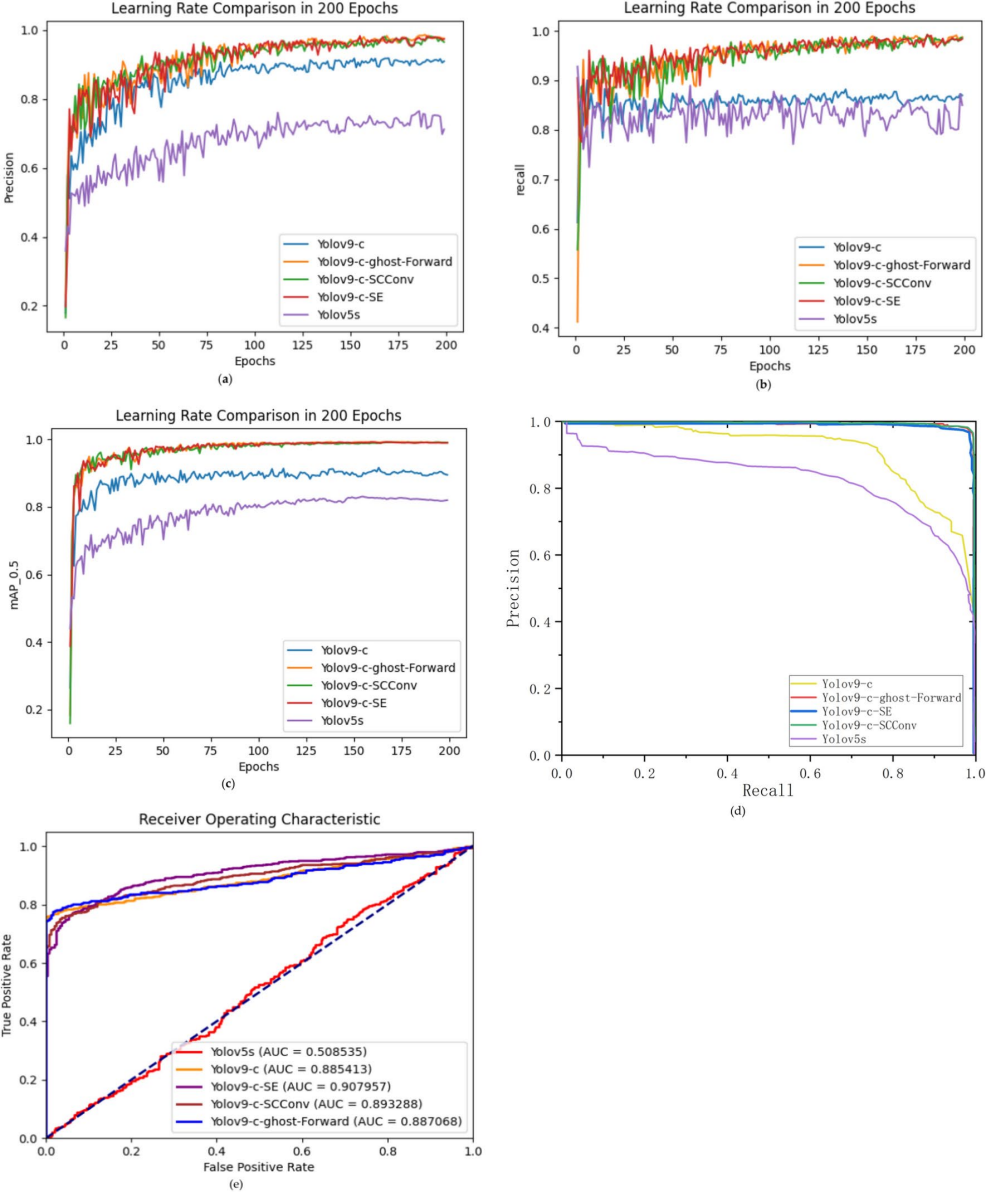
Comparison of the performances. (**a**) precision growth curves, (**b**) recall growth curves for the five models, (**c**) mAP0.5 growth curve comparison, (**d**) P-R curve comparison, and (**e**) ROC curve comparison.

According to our investigation, the Yolov5s, Yolov9-c, Yolov9-c-SE, Yolov9-c-SCConv, and Yolov9-c-ghost-Forward models were trained. Among them, the Yolov5s-7.0 model was trained using an unprocessed dataset with a network depth of 214 layers and 7,025,023 parameters. The performance indicators for these five models are presented in Fig. 18.

As shown in Fig. 18, the training results of Yolov5s are relatively poor due to the limitation of the model itself and the lack of excellent data images. After switching the model and applying data augmentation, Yolov9-c can achieve a better performance, but still not as good as the ECA-Improved-Yolov5s-Mobilenet model proposed by Xia et al. in 2024^32^. The improved Yolov9-c-SE and Yolov9-c-SCConv models have more parameters than Yolov9-c, the precision is somewhat improved, and the recall can reach nearly 98.8% or more. In contrast, Yolov9-c-ghost-Forward can show excellent performance with a slight increase in the number of parameters, with a precision of 98.6% and a recall of 98.6%.

The mAP0.5 values of the improved Yolov9-c-SE, Yolov9-c-SCConv, and Yolov9-c-Ghost-Forward models are all stabilized at around 99%, which is an excellent figure.

The P-R curves of the Yolov9-c-SE, Yolov9-c-SCConv, and Yolov9-c-ghost-Forward models can completely wrap around the P-R curves of the Yolov5s and Yolov9-c models with similar trajectories. Thus, it can be concluded that the performance of these three improved models is superior to that of Yolov5s and Yolov9-c models.

When comparing AUC values and F1 scores, the Yolov9-c-SE, Yolov9-c-SCConv, and Yolov9-c-ghost-Forward models consistently outperform the Yolov9-c model, indicating that the experiments in this study have produced meaningful results.

Subsequently, after analyzing the above findings, the best-combined detection results for each model were selected in this study and compared with the results of the ECA-Improved-Yolov5s-Mobilenet model proposed by Xia in 2024^32^ are compared with the outcomes of experiments as shown in Table 7. The Yolov9-c-ghost-Forward model performs well in soybean seed surface defect detection, achieving 99.2% mAP0.5 and 98.6% accuracy, outperforming Yolov9-c, Yolov9-c-SE, and Yolov9-c-SCConv. This is mainly because the Yolov9-c-ghost-Forward model effectively improves feature extraction capability and reduces computational complexity, all while enhancing recognition accuracy. The GhostConv module introduces a significant improvement by enhancing feature extraction and reducing complexity, while balancing lightweighting and performance. The model’s lightweight design is also advantageous for use in agricultural fields with limited computational resources. Additionally, the GhostConv module’s ability to suppress image noise provides a distinct advantage in improving detection accuracy for surface defects in soybean seeds. Techniques such as grayscale conversion, filter processing, image segmentation, and morphological manipulation effectively reduce noise, further improving detection performance.

**Table 7:** Model testing results.

Model name	Accuracy (%)	Recall (%)	mAP0.5 (%)	F1 Score	AUC
ECA-Improved-Yolov5s-Mobilenet^32^	92.8	98.9	95.5	–	–
Yolov5s	78.2	77.3	82.2	0.88	0.51
Yolov9-c	90.8	86.8	89.6	0.95	0.89
Yolov9-c-SE	95.3	98.8	98.9	0.92	0.91
Yolov9-c-SCConv	95.8	99.0	98.9	0.95	0.89
Yolov9-c-ghost-Forward	98.6	98.6	99.2	0.96	0.89

While the Yolov9-c-ghost-Forward model excels in accuracy, it may experience relatively higher computational overhead and slower inference speed. In contrast, lightweight models like Yolov5s and ECA-Improved-Yolov5s-Mobilenet may offer better performance in terms of computational resource consumption, but there is still a gap in terms of accuracy. This difference in performance can be attributed to two main factors: first, the latest Yolov9 model has undergone numerous improvements and optimizations compared to Yolov5, resulting in superior performance. Second, the network layers of Yolov9 are deeper and more complex than those of Yolov5, which explains why the improved Yolov9-c-ghost-Forward model based on Yolov9 outperforms both Yolov5s and ECA-Improved-Yolov5s-Mobilenet.

Moreover, the current research focuses on improving a model for binary classification, which cannot identify seeds with different types of damage. This limitation results in some seeds being deemed unusable. For example, using broken or epidermally damaged soybeans as seeds may decrease yield but does not significantly affect the production of livestock feed. However, using diseased or heavily damaged seeds could pose a threat to the livestock’s health. On the other hand, multi-classification problems tend to be more complex, requiring further study and exploration to advance the relevant science and technology. Additionally, although this study has improved the model’s lightweight while ensuring recognition accuracy, many remote farms still lack access to high-computing power equipment for automatic screening. Therefore, balancing the model’s lightweight and accuracy will be the focus of future improvements.

Considering the extensive production of soybeans, where a 1% error can result in the loss of millions of tons of soybeans, these two models do not meet the expectations of this study. In contrast, only Yolov9-c-ghost-Forward can manage to improve all the data, and Yolov9-c-ghost-Forward only increases the network depth by 0.7% and the number of parameters by 2% relative to Yolov9-c. Therefore, the improved Yolov9-c-ghost-Forward model with the ghost module may be an ideal solution in practical applications.

## Conclusions

In this study, the Yolov9-c-ghost-Forward neural network model for soybean seed surface defect detection was crafted based on the Yolov9-c architecture and enhanced by incorporating GhostConv, a lightweight convolutional module from GhostNetThis enhancement improves the recognition of soybean seed images through techniques such as grayscale conversion, filtering, image segmentation, and morphological operations, and significantly reduces noise, thereby separating the soybean seeds from the original images. The Yolov9-c-ghost-Forward model achieved the highest mAP0.5 of 99.2% compared to Yolov9-c, Yolov9-c-SE and Yolov9-c-SCConv. In addition, the Yolov9-c-ghost-Forward model showed a significant improvement in accuracy of 98.6% compared to the Yolov9-c, Yolov9-c-SE, and Yolov9-c-SCConv models. We will further improve the Yolov9-c-ghost-Forward model to reduce its size while ensuring its high accuracy and realizing the model’s lightweight.

## Data Availability

The data used in this article can be downloaded from the following link https://www.kaggle.com/datasets/warcoder/soyabean-seeds.
